# Abnormal characteristics of inferior vena cava and abdominal aorta among neonates with early onset septic shock

**DOI:** 10.1186/s13052-024-01829-0

**Published:** 2025-01-29

**Authors:** Lanlan Mi, Yiman Liu, Fei Bei, Jianhua Sun, Jun Bu, Yuqi Zhang, Weiwei Guo

**Affiliations:** 1 Department of Neonatology, Shanghai Children’s Medical Center, School of Medicine, Shanghai Jiao tong University, Shanghai, China; 2 Department of Pediatric Cardiology, Shanghai Children’s Medical Center, School of Medicine, Shanghai Jiao tong University, Shanghai, China

**Keywords:** Neonatal hemodynamics, Inferior vena cava, Inferior vena cava collapsibility index, Inferior vena cava to abdominal aorta ratio, Early onset septic shock

## Abstract

**Background:**

The variety of shocks in neonates, if not recognized and treated immediately, is a major cause for fatality. The use of echocardiography may improve assessment and treatment, but its reference values across gestational age (GA) and birth weight (BW) are lacking. To address the information gap, this study aimed at correlating GA and BW of newborns with nonhemodynamic abnormalities, and at evaluating the usefulness of such reference values in neonates with early onset septic (EOS) -shock.

**Methods:**

A total of 200 normal newborns were enrolled as controls and subdivided into groups based on GA, BW, days of age, and patent ductus arteriosus (PDA). Echocardiography was used to document inferior vena cava diameter (IVC), inferior vena cava collapsibility index (IVC-CI), and inferior vena cava to abdominal aorta ratio (IVC/AO). In addition, 18 neonates with EOS shock were recruited and evaluated using echocardiography.

**Results:**

Among the control newborns, IVC and AO were significantly increased with GA and BW (*P* < 0.05) but IVC-CI and IVC/AO did not correlate with GA, BW, day of age, and PDA. Compared to the control group, the EOS-shock group had significantly decreased IVC and IVC/AO, and increased IVC-CI (*P* < 0.05). The cut-off values for indicating EOS-shock were > 34.15% for IVC-CI, < 47.58% for IVCmin/AO, and < 66.11% for IVCmax/AO.

**Conclusions:**

The IVC-CI, IVCmin/AO, and IVCmax/AO indices are applicable to all neonates. Although the number of neonates with EOS-shock in our study is small, the cut-off values showed usefulness for diagnosis. Further research is needed to determine the application of the indices in a larger population and among other populations, especially for clinical application in treatment of shock among neonates.

## Introduction

In pre-term infants, shock typically causes circulatory dysfunction and neurodevelopmental impairment, with fatal consequences. Immediate medical attention is therefore critical for good prognosis [1] and fluid resuscitation is the first step for hemodynamically unstable patients [2]. Echocardiography is useful for providing guidance for shock management in adults [3] and children [4], but limited for neonates [5]. Nevertheless, some measurements are promising. For example, inferior vena cava diameter (IVC) via point-of-care ultrasound appears to correlate with patient’s volume status [6]. IVC collapsibility index (IVC-CI) has been shown to be elevated in septic shock neonates versus stable controls [5]. Inferior vena cava to abdominal aorta ratio (IVC/AO) has been invastigated to guide pediatric fluid resuscitation [4]. These measurements have, however, not been systematically evaluated, especially for pre-term infants.

This study aimed to determine IVC, IVC-CI and IVC/AO by echocardiography in newborns with different gestational ages and weights, investigate their correlations, and to apply these measurements in newborns with early onset septic (EOS) -shock.

## Patients and methods

This cross-sectional analytical study was approved by the hospital ethics committee and conducted at the neonatal intensive care unit of the Shanghai Children’s Medical Center from September 2022 to March 2023. Written informed consents were obtained from the parents or guardians for the study subjects. Those with 48 h to one week of age, and without sepsis or shock (control group) were recruited because blood pressure and vascular resistance increase while cardiac output and index decrease in the first 48 h [7]. Septic shock patients (refer to those with early-onset sepsis and shock, EOS-shock group) were enrolled during the study period. Early-onset sepsis was defined as hospitalization with at least 1 laboratory criterion and either respiratory distress or at least 2 other clinical criteria [8]: (1) Heart rate > 180 per min, (2) decrease in blood pressure (mean arterial pressure (MAP) < 30 mm of Hg or < MAP < 5th centile for the gestational age or systolic blood pressure < 2 SD for age), (3) oliguria < 0.5 ml/kg/h for preceding 6 h, (4) CRT(capillary refilling time) > 3 s, (5) central to peripheral temperature difference > 3 °C, (6) metabolic acidosis (base excess [BE] > − 5 or lactate > 2 times upper normal). Shock was defined as having at least 2 of the 6 criteria [9]. Exclusion criteria were admission after 24 h of birth, congenital heart disease (e.g., mild to severe left-to-right intracardiac shunt disease and valve disease), pulmonary hypertension, obvious right heart failure, complex cyanotic heart disease (to avoid the impact on right heart and inferior vena cava hemodynamics), high-frequency ventilation (IVC visualization is difficult), congenital multiple malformations and death within 7 days. Demographic, clinical, and hemodynamic data were collected, including patient gender, gestational age, birth weight, days of age, delivery mode, Apgar 1 min, Apgar 5 min, mother’s situation (%, thyroid dysfunction, hypertension, gestational diabetes mellitus (GDM), immune diseases, premature rupture of membrane (PROM) ≥ 18 h, stained amniotic fluid, prenatal antibiotics, pathogenic positivity), blood pressure (BP) (systolic blood pressure, diastolic blood pressure and mean blood pressure), heart rate (HR), left ventricular ejection fraction (LVEF), and lactate etc.

### IVC-CI and AO

**Fig. 1:**
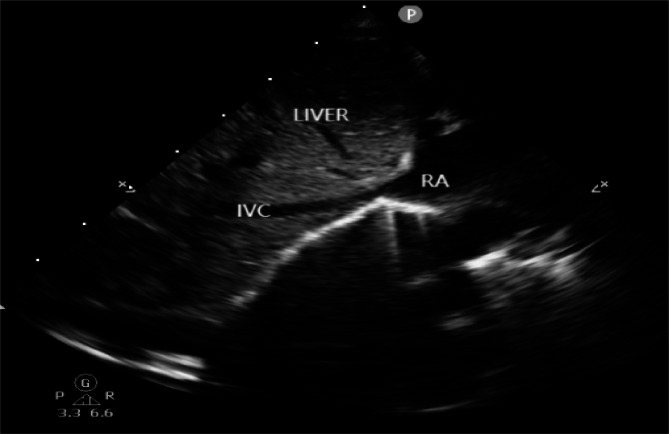
2-D ultrasound showing IVC entering the right atrium

**Fig. 2:**
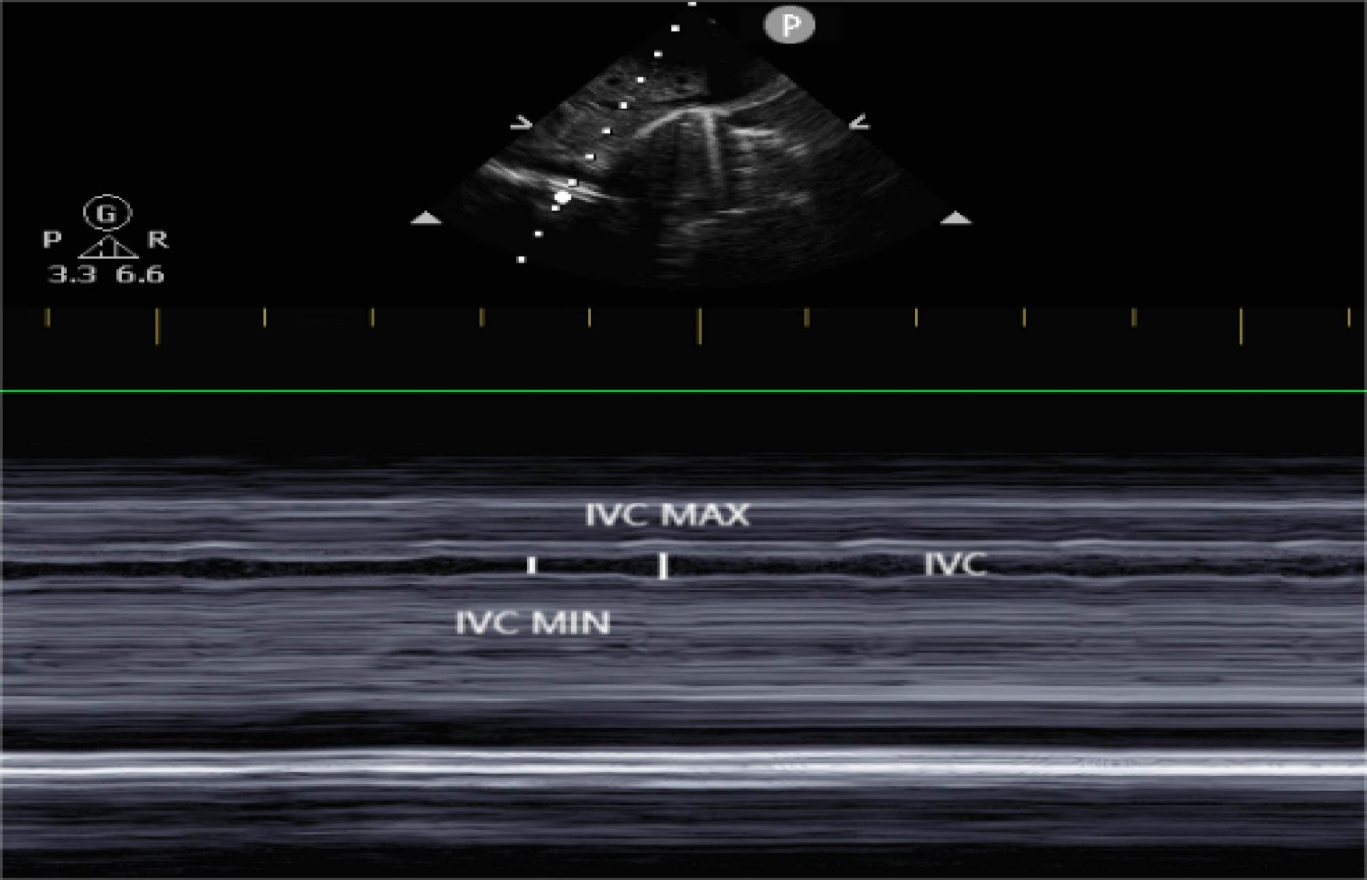
2-D ultrasound showing IVCmin and IVCmax

With patients in the supine position, IVC and AO diameter were measured using color Doppler ultrasound (Philips CX50, probe S8-1 with frequency of 5 MHZ) in the M-mode. The probe was placed on the lower right of the xiphoid process to visualize the IVC long axis near the hepatic vein inflow. The IVC diameter was measured 0.5–1.0 cm below the hepatic vein inflow in M-mode (shown in Fig. 1). Three measurements of the maximum and minimum diameters were taken and averaged (shown in Fig. 2). IVC-CI = (maximum IVC diameter – minimum IVC diameter) / maximum IVC diameter. According to the US echocardiography guidelines, the subxiphoid long-axis views of the abdominal aorta were used to measure the internal diameter of the abdominal aorta during diastole.

### Statistical analyses

Statistical analyses were performed using Prism (Version 9; GraphPad, USA). It was sufficient to include 200 newborns in this study to establish a reference range for the study indicators [10]. Due to the scarcity of sources of newborns with early-onset septic shock, a total of 18 patients who met the inclusion criteria were collected during this study. Paired t-test analysis was used to analyze the same indicators collected by two observers. Logistic regression was used to analyze influencing factors for IVC, AO, IVC-CI, and IVC/AO. Linear regression was used to evaluate relationships between gestational age, birth weight, and days of age with hemodynamic parameter. Paired t-tests were used to compare PDA group and non-PDA group (the same patient before and after PDA closure), and non-paired t-tests compared the control group and shock group. The ROC curve analysis was used to accurately assess the ability of IVC-CI, IVCmin/AO, and IVCmax/AO to predict shock, with cut-off identified by the Youden index. Measurement data are presented as mean ± SEM, and categorical data as number (percentage) [n (%)]. *P* < 0. 05 was considered statistically significant.

## Results

**Fig. 3:**
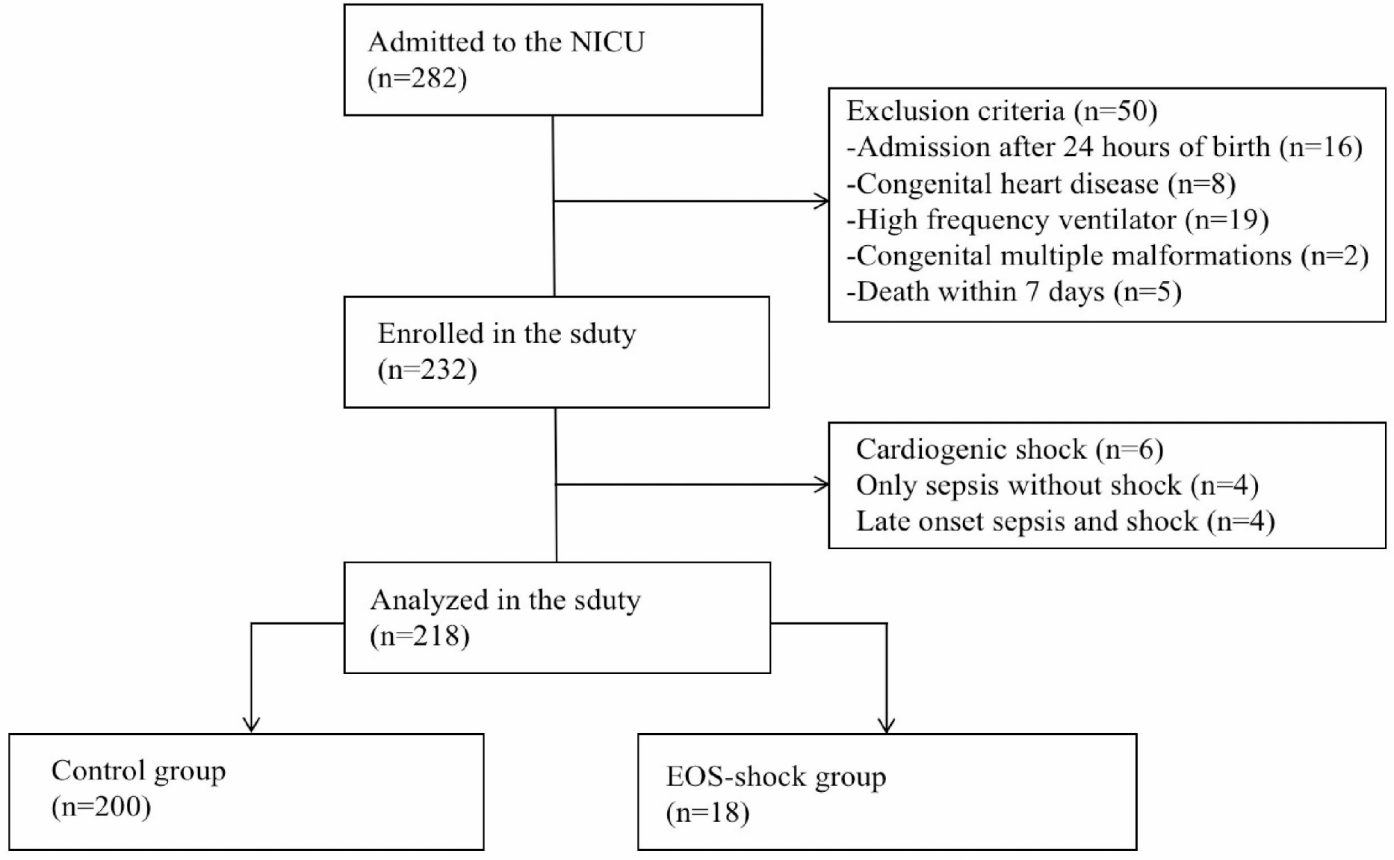
Flowchat of the sduty. EOS-shock group: early onset septic shock group

A total of 282 neonates were assessed for eligibility (shown in Figs. 3) and 218 neonates were selected based on inclusion and exclusion criteria: 200 in the control group and 18 in the early onset septic shock group. There was no significant difference between inter-observer measurements (*p* < 0.05).

Among the 200 controls, 10 were born at gestational age of less than 29 weeks, 68 at 29 to 33 + 6 weeks, 82 at 34 to 36 + 6 weeks, and 40 at term. Eight neonates had birth weights less than 1000 g, 19 between 1000 g and 1499 g, 117 between 1500 g and 2499 g, and 56 more than 2500 g. IVCmin, IVCmax and AO increased significantly with advancing gestational ages and higher birth weights (*p* < 0.0001 for all). In contrast, the IVC-CI and IVC/AO (IVCmin/AO and IVCmax/AO) did not demonstrate significant correlations with gestational ages or birth weights.

These findings provide reference ranges and linear regression equations for inferior vena cava measurements according to gestational age and birth weight categories in neonates without hemodynamic disturbances (Table 1).

**Table 1:** IVC, IVC-CI, and IVC/AO of neonates with different gestational ages and birth weight

Index	Age(w)								Birth weights(g)						
	< 29w	29–33 ^+ 6^w	34–36 ^+ 6^w	≥ 37w	r	*p* value	Linear regression equation		<1000 g	1000–1499 g	1500–2499 g	≥ 2500 g	r	*p* value	Linear regression equation
	*n* = 10	*n* = 68	*n* = 82	*n* = 40					*n* = 8	*n* = 19	*n* = 117	*n* = 56			
IVCmin(mm)	2.3(±0.5)	2.6(±0.5)	2.7(±0.5)	3.3(±0.5)	0.5071	<0.0001	Y = 0.009105*X-0.03673		2.4(±0.5)	2.4(±0.5)	2.6(±0.5)	3.2(±0.5)	0.5621	<0.0001	Y = 3.878e-005*X + 0.1913
IVCmax(mm)	3.1(±0.6)	3.5(±0.6)	3.7(±0.6)	4.4(±0.6)	0.5375	<0.0001	Y = 0.01178*X-0.03450		3.0(±0.6)	3.3(±0.6)	3.6(±0.6)	4.3(±0.6)	0.6446	<0.0001	Y = 5.429e-005*X + 0.2514
IVC-CI(%)	31.1%(±5.9%)	30.0%(±6.3%)	31.2%(±6.5%)	29.5%(±5.7%)	NS	NS	NS		27.64%(±6.13%)	31.39%(±5.99%)	30.51%(±6.41%)	30.35%(±6.14%)	NS	NS	NS
AO(cm)	4.0(±0.8)	4.8(±0.7)	5.0(±0.7)	6.0(±0.7)	0.6785	<0.0001	Y = 0.01740*X-0.09128		4.0(±0.7)	4.4(±0.8)	4.9(±0.7)	5.9(±0.7)	0.7703	<0.0001	Y = 7.589e-005*X + 0.3404
IVCmin/AO(%)	58.5%(±8.3%)	54.6%(±8.0%)	54.3%(±8.0%)	54.9%(±7.4%)	NS	NS	NS		59.9(±8.3%)	55.8%(±8.2%)	54.0%(±8.0%)	55.0%(±8.0%)	NS	NS	NS
IVCmax/AO(%)	78.4%(±9.1%)	72.8%(±9.3%)	73.7%(±9.5%)	72.9%(±8.7%)	NS	NS	NS		76.9%(±8.9%)	75.5%(±9.1%)	72.7%(±9.4%)	73.9%(±9.2%)	NS	NS	NS

NS: *P* > 0.05

Among the 200 newborns in the control group, 67 were under 3 days old, 68 were between 3 and 5 days old, and 67 were between 5 and 7 days old; 10 individuals with PDA were within one week after birth and closed within two weeks. The IVC-CI and IVC/AO (IVCmin/AO and IVCmax/AO) were not related to postnatal days of age, nor were they related to the presence of PDA (Not hs-PDA) (Table 2).

**Table 2:** IVC-CI, IVCmin/AO and IVCmax/AO of neonates with different day ages and with PDA or not

Index	Age(days)					PDA or Not		
	< 3 days (*n* = 67)	3–5 days (*n* = 68)	5–7 days (*n* = 67)	*p* value		PDA	Non-PDA	*p* value
IVC-CI(%)	30.9%(±5.5%)	30.9%(±7.7%)	29.3%(±5.8%)	NS		32.5%(±6.6%)	30.4%(±5.9%)	NS
IVCmin/AO(%)	53.7%(±7.1%)	53.1%(±8.0%)	56.8%(±8.7%)	NS		54.4%(±8.0%)	56.7%(±6.8%)	NS
IVCmax/AO(%)	72.5%(±8.2%)	72.6%(±10.2%)	74.9%(±9.4%)	NS		71.4%(±10.1%)	74.5%(±8.0%)	NS

NS: *P* > 0.05

Comparing the baseline characteristics between the 200 controls and the 18 EOS-shock newborns, it was found that, except for birth weight, other general characteristics were not statistically significant (Table 3).

**Table 3:** Comparison of characteristics between the control group and the septic shock group

Characteristics	C group	S group	*p* value
Gestational age (w)	34.6(± 2.9)	35.7(± 2.3)	0.2701
Birth weight (g)	2224(± 750)	2670(± 640)	0.0201
Gender Male (%)	112(56)	12(54.5)	0.8968
Delivery mode Vaginal delivery (%)	45(22.5)	5(22.7)	0.9808
Apgar 1 min	8.6(± 1.2)	9.3(± 1.8)	0.0561
Apgar 5 min	9.3(± 0.7)	9.5(± 0.9)	0.3164
Thyroid dysfunction (%)	48(24)	2(9.1)	0.1131
Hypertension (%)	18(9)	1(4.5)	0.4806
GDM (%)	38(19)	4(18.2)	0.9263
Immune diseases (%)	25(12.5)	1(4.5)	0.2728
PROM ≥ 18 h (%)	9(4.5)	2(9.1)	0.3485
Stained amniotic fluid (%)	10(5)	1(5)	0.9261
Prenatal antibiotics (%)	40(20)	3(14)	0.4757
Pathogenic positivity (%)	30(15)	4(18)	0.6957

C group: control group; S group: early onset septic shock group; GDM: Gestational diabetes mellitus; PROM: Premature rupture of membrane

The IVC-CI, IVCmin/AO, and IVCmax/AO of the control group were 30.45 ± 6.47%, 54.27 ± 9.49%, and 73.12 ± 9.34%, respectively, while those of the EOS shock group were 39.64 ± 9.27%, 41.61 ± 8.00%, and 64.96 ± 10.96%, respectively. In the similar comparison, the EOS shock group had increased IVC-CI but decreased IVCmin/AO and IVCmax/AO (*p* < 0.0001). The cut-off values for the EOS shock group were > 34.15% for IVC-CI, < 47.58% for IVCmin/AO, and < 66.11% for IVCmax/AO (Table 4).

**Table 4:** Comparison of IVC-CI, IVCmin/AO and IVCmax/AO between the control group and septic shock group, along with corresponding cut-off values

Index	C group(%)	S group(%)	Significance (*p* value)	Cut-off (%)	Sensitivity(%)	Specificity(%)	Youden index
IVC-CI	30.5 ± 6.5	39.6 ± 9.3	< 0.0001	> 34.2	82.4	74.8	0.68
IVCmin/AO	54.3 ± 9.5	41.6 ± 8.0	< 0.0001	< 47.6	88.2	81.3	0.68
IVCmax/AO	73.1 ± ± 9.3	65.0 ± 11.0	< 0.0001	< 66.1	81.3	76.3	0.50

C group: control group; S group: early onset septic shock group

## Discussion

Even with clinical recognition and targeted hemodynamic resuscitation [11–13], the mortality rate for neonates with septic shock was as high as 34% [14]. The high burden was likely due to the non-precise clinical evaluation [15, 16] and invasive risk from the hemodynamic monitoring [17]. On the other hand, ECHO showed good consistency with invasive hemodynamic methods such as pulse index contour cardiac output (PiCCO) [18] and central venous pressure (CVP) [19].

According to the guidelines from the American Echocardiography Association, during forced breathing for adults, the width of the IVC and the IVC-CI should correspond to CVP, indicating the amount of fluid should guide clinical decision-making [20]. Therefore, the combination of IVC and IVC-CI can improve the accuracy of blood volume assessment. In a study of 70 children aged 1 month to 12 years, the results show a negative correlation between IVC-CI and CVP. IVC-CI > 50% corresponded to CVP < 8 cmH2O, indicating insufficient blood volume and usefulness of IVC-CI to evaluate the blood volume status of critically ill pediatric patients [21]. These indicators show their application for management of pediatric shock patients [22, 23], but without evidence for neonates. Our study fulfills the knowledge gap by providing reference values for IVC parameters across gestational ages and birth weights among stable neonates.

In this study, IVCmin, IVCmax, and AO were collected by a cardiac ultrasound doctor and a NICU doctor, and there were no statistically significant differences between them. In a study using adults, there was intra- and inter-observer accuracy in measuring IVC by ECHO [24]. This indicates that the parameters can serve as important indicators for evaluating hemodynamics in critically ill patients. Our results provide normal reference ranges for IVCmin, IVCmax, IVC-CI, IVCmin/AO, and IVCmax/AO stratified by gestational maturity and birth weight in hemodynamically stable neonates (the control group). IVC diameter and AO increased with greater maturity and size, while IVC-CI and IVC/AO were similar across groups. Another study in neonates shows that there was a good negative correlation between IVC-CI and CVP in mechanically ventilated patients, but no correlation with gestational age and weight. IVCmax and IVCmin were not correlated with CVP, but had a good positive correlation with gestational age and weight [25]. A study in healthy children also shows that age, height, and weight were positively correlated with IVCmax and IVCmin, while IVC-CI was not significantly correlated with age, height, and weigh [26]. These are consistent with the results of our study. In addition, IVC-CI, IVCmin/AO, and IVCmax/AO did not change with the days of increased age, and the presence of the PDA (Not hsPDA) did not affect them. In autonomous breathing, the IVC contracts during inhalation and expands during exhalation. However, during positive pressure mechanical ventilation, the intrathoracic pressure and right atrial pressure increase during inhalation, and the amount of blood flowing back from veins to the right atrium decreases, affecting the diameter of the IVC. The IVC expands during inhalation and contracts during exhalation. Therefore, the measurement of IVCmax and IVCmin in patients with positive pressure mechanical ventilation was opposite to the normal breathing state, that is, IVCmax was measured during inhalation and IVCmin was measured during exhalation [27]. Another study show that IVC-CI was not affected by whether patients were breathing spontaneously or were mechanically ventilated [28]. Intra-abdominal hypertension had no effect on IVC-CI but reduced IVCmax, and large IVCmax with no collapse, that is, not hypovolemic [29]. The above information indicates that the IVC-CI value did not depend on individual’s physical parameters and breathing patterns, but rather on blood volume status, making it a good indicator for evaluating blood volume. In summary, IVC-CI, IVCmin/AO, and IVCmax/AO were not affected by gender, gestational age, and birth weight. Therefore, newborns were organized into one age group.

The target range of IVC-CI was usually between 20% and 50% [28, 30], which is different from the approximately 18–40% range of IVC-CI in newborns in this study. This may be because neonates normally have faster respiratory rate and lower respiratory amplitude than adults or children. A report on 23 healthy premature infants with a gestational age of 30.9 ± 2.9 weeks and a birth weight of 1146 (966, 1460) g shows that IVC-CI fluctuated between 15% and 24%, with an average of 20% [31]. Another report shows measured IVC-CI values of 12–46% (P10 to P90) in 25 healthy full-term infants with an average weight of 3425 g [32]. There is a certain deviation from the results of this study, which may be explained by different sample sizes. Nonetheless, these values provide context for interpreting IVC measurements in hypotensive states.

Except for birth weight, the general characteristics of the EOS-shock group were not statistically significant compared to the control group. As shown above, IVC-CI, IVCmin/AO, and IVCmax/AO were not affected by weight, therefore the two groups were comparable. Our observations show that IVC and IVC/AO declined markedly while IVC-CI rose in neonates with EOS-shock compared to stable controls across etiologies. These findings concur with adult and pediatric studies showing IVC narrowed and collapsed to a greater degree in shock [33, 34]. A study was reported on comparing the levels of 5 preload indicators between a septic shock and a healthy control groups of 46 premature infants [31]. The results show that only IVC-CI was significantly increased in the septic shock group, which is consistent with the results of our study.

A report described IVC collapsibility > 40% as 93% sensitive and 100% specific for hypovolemic shock pediatric patients [23]. A prospective longitudinal study involved 66 newborns with low blood volume (CVP < 5 cmH2O), 22 newborns with normal blood volume (CVP 5–8 cmH2O), and 34 newborns with high blood volume (CVP > 8 cmH2O) [35]. The results show that the sensitivity of predicting low blood volume at a cut-off value of 55% for IVC-CI was 87.9%, the specificity was 82.0%, and the sensitivity of predicting high blood volume at a cut-off value of 20% was 91.1%, the specificity was 83.2%. IVC-CI was significantly and negatively correlated with CVP, suggesting that IVC-CI can be used to guide fluid resuscitation and the application of vasoactive drugs in neonatal shock. In our study, IVC-CI > 34.15% had a sensitivity of 82.35% and a specificity of 74.75% for EOS-septic shock. The lower IVC-CI may be explained by the shallower and faster breathing of newborns, while the lower sensitivity and specificity may be explained by the insufficient number of shock patients in newborns. A previous study shows the optimal cut-off value of IVC/AO was 0.675 in neonates [4], which was similar to IVCmax/AO < 66.11% in our study. Our data also provides an optimal cutoff value of IVCmin/AO < 47.58%, which can be combined with the cutoff values of IVC-CI and IVCmax/AO to jointly warn of EOS-septic shock. Our findings reinforce the potential diagnostic utility of IVC metrics for compromised neonatal circulation.

## Conclusions

Our study provides novel information but also has limitations, including small sample size and single center data. Larger scale and multicenter studies (like our ongoing study) should confirm the universal application in newborn populations. Our current observations will also be enhanced by our new research on changes of IVC-CI, IVCmin/AO and IVCmax/AO in different fluid volumes before and after resuscitation in shock, as well as their relationships with CVP. Nevertheless, our research findings demonstrate clinical practicality of IVC ultrasound monitoring in evaluating and managing neonatal EOS-shock. The reference values from stable newborns provide a background for interpreting measurements. IVC-CI, IVCmin/AO and IVCmax/AO can be used to determine the presence of EOS-shock from a capacity perspective. Further research should evaluate the integration of these tools with neonatal shock diagnosis and treatment to improve outcomes.

## Data Availability

All data generated or analyzed during this study are included in this article. Further inquiries can be directed to the corresponding author.

## Abbreviations

IVC: Inferior vena cava diameter
IVC-CI: Inferior vena cava collapsibility index
IVC/AO: Inferior vena cava to abdominal aorta ratio
CVP: Central venous pressure
GA: Gestational age
BW: Body weight
GDM: Gestational diabetes mellitus
PROM: Premature rupture of membrane
ECHO: Echocardiography
HR: Heart rate
SPB: Systolic blood pressure
DBP: Diastolic blood pressure
MAP: Mean arterial pressure
